# Temperature and Time Requirements for Controlling Bed Bugs (*Cimex lectularius*) under Commercial Heat Treatment Conditions

**DOI:** 10.3390/insects2030412

**Published:** 2011-08-29

**Authors:** Stephen A. Kells, Michael J. Goblirsch

**Affiliations:** Department of Entomology, University of Minnesota, 219 Hodson Hall, 1980 Folwell Ave., St. Paul, MN 55108, USA; E-Mail: goblirmj@umn.edu

**Keywords:** Hemiptera, lethal temperature, lethal time, logistic regression, Probit

## Abstract

Developing effective alternative approaches for disinfesting bed bugs from residential spaces requires a balance between obtaining complete insect mortality, while minimizing costs and energy consumption. One method of disinfestation is the application of lethal high temperatures directly to rooms and contents within a structure (termed whole-room heat treatments). However, temperature and time parameters for efficacy in whole-room heat treatments are unknown given the slower rate of temperature increase and the probable variability of end-point temperatures within a treated room. The objective of these experiments was to explore requirements to produce maximum mortality from heat exposure using conditions that are more characteristic of whole-room heat treatments. Bed bugs were exposed in an acute lethal temperature (LTemp) trial, or time trials at sub-acute lethal temperatures (LTime). The lethal temperature (LTemp_99_) for adults was 48.3 °C, while LTemp_99_ for eggs was 54.8 °C. Adult bed bugs exposed to 45 °C had a LTime_99_ of 94.8 min, while eggs survived 7 h at 45 °C and only 71.5 min at 48 °C. We discuss differences in exposure methodologies, potential reasons why bed bugs can withstand higher temperatures and future directions for research.

## 1. Introduction

The bed bug, *Cimex lectularius* L. (Hemiptera: Cimicidae), has maintained a close association with humans and other warm-blooded animals since ancient times [1]. Encounters with bed bugs are becoming very common due to the recent resurgence in infestations in North America and other locations [2-4]. Until recently, insecticides were the principal means of eliminating bed bugs from a site. However, resistance to insecticides [5] and issues with insecticide efficacy [6] now require that bed bug infestations undergo multiple insecticide applications. Restrictions imposed by insecticide labels and concerns of liability, particularly with applications to mattresses, have reduced the extent of insecticide applications made against this pest. Even with thorough insecticide applications, there are items and locations within homes and apartments that will require other disinfestation methods. Occupants are often responsible for disinfesting items that cannot be treated with an insecticide; however, this practice increases the potential for treatment failure because the occupants may not employ sufficient attention to detail required for disinfesting articles.

Use of high temperatures against bed bug infestations is not a novel approach, but recent development of commercial equipment has allowed this method to become mainstream [7,8]. Heat treatments may occur within containers such as insulated boxes [9] and heated shipping trailers, or heat may be delivered directly to rooms and contents within a structure (termed whole-room heat treatments). Regardless of the method used, there are advantages to utilizing heat treatments as part of an integrated pest management (IPM) strategy. Such advantages include: reduced usage of insecticides in living spaces, more immediate efficacy, the ability to treat a diversity of articles, and a reduction in effort to process different items such as infested laundry and electronics.

Deployment of heat for treatment of living spaces presents some challenges that must be considered. Principal considerations include the temperature and time characteristics necessary to achieve lethal conditions. Since heat treatments do not provide residual control, a goal of 100% efficacy with the primary treatment would eliminate the need for subsequent applications. However, excessive temperatures or hold times during treatment can increase operation costs through energy consumption, equipment use and labor; and may increase the risk of damage to furniture and other articles. The current temperature range targeted by companies providing heat treatments is 45 to 52 °C; however, there may be places in the heated area above and below this range. Often the air-space within a heat treated room may approach 55 to 65 °C to ensure efficient mass transfer of heat. Consequently, the final temperature that bed bugs will be exposed to will depend on insulative and thermal mass properties of the heated materials. Providing precise temperature and hold time parameters will ensure maximal efficacy, minimize the risk of damage to occupant property and keep operational costs low.

The lethal temperature for bed bugs was previously reported as 45 °C for eggs and 44 °C for adults during a 1-h exposure [10]. Mellanby [10] reported that a 24-h exposure at 40 °C was sufficient to kill all life stages. Pereira *et al.* [9] indicated that mortality of adult bed bugs started at 41 °C with an exposure time of 100 min and this time decreased to 1 min at 49 °C. However, this threshold was determined through rapid exposure to high temperatures with a rate of temperature increase that is considerably different to the application of heat in a whole-room treatment. Slower rates of heating, as in whole-room applications, may permit bed bugs to respond physiologically [11] or behaviorally [12]. Also, previous temperatures encountered by insects may allow for acclimation that can raise the lethal temperature and time thresholds for insects [13]. The objective of this study was to determine the temperature and exposure time necessary to produce maximal mortality from heat using conditions similar to a whole-room treatment. Our results show that successful control of bed bugs via heat treatments must employ temperatures and exposure times that are substantially higher than values previously cited in the literature.

## 2. Experimental Section

### 2.1. Source of Insects

Bed bugs were collected from six commercial properties located in Minnesota, Wisconsin, Florida and New Jersey and were combined to form the ECL-05 “field” colony in 2005 [14]. Bed bugs from the field colony were cultured in 473 mL glass jars containing several folded filter papers for harborage and covered with fine mesh for ventilation. The colony was maintained at 27 °C, 70% RH and 16:8 L:D photoperiod in a Controlled Environment Chamber (CEC) (Percival Scientific, Inc.; Perry, IA). Bed bugs were fed whole, heparinized human blood through an artificial membrane system similar to Montes *et al.* [15]. Human blood was procured from expired stocks purchased from the American Red Cross (St. Paul, MN).

### 2.2. Adult Bed Bugs

For experiments using adults, groups of fed or unfed bed bugs were placed separately into 6 mL plastic vials with 10 individuals per vial and a total of 72 vials (719 adults). Each vial contained a 1 × 3 cm filter paper strip for harborage. The vial had a cap with a 4-mm hole blocked with filter paper for ventilation. Bed bugs categorized as “fed” were provided blood *ad libitum* and used in experiments within 24 h of feeding. Bed bugs that were categorized as “unfed” had not been offered a blood meal at least 14 d prior to use in experiments. Vials containing adult bed bugs were maintained under conditions identical to the colony until use.

### 2.3. Egg Collection

At least 10 engorged adult bed bugs per vial were placed into 6 mL plastic vials containing several 1 × 3 cm filter paper strips for harborage and oviposition. Sex ratios in the vials were heavily skewed toward females. Vials were maintained under conditions identical to those of the colony. Viable eggs, identified by their smooth and white appearance, were collected after 5 d and immediately used in experiments. At least 10 eggs were placed into each vial and there were 32 vials (370 eggs).

### 2.4. Heating Devices

A programmable CEC was used to increase the temperature at a constant rate of 0.06 °C/min (3.6 °C/h) that allowed a temperature increase from 30 °C to 50 °C. Rate of temperature increase was based on preliminary measurements from monitoring a house and a two-bedroom apartment during previous treatments for bed bug infestations. The chamber lights remained off during the trials. Due to limitations on the maximum temperature output of the CEC, the oven of a gas chromatograph (GC) (6890N, Agilent Technologies, Inc.; Santa Clara, CA) was employed for experiments that required temperatures in excess of 50 °C. Also, the small volume and more precise temperature control of the GC oven allowed it to be used in the lethal temperature experiments. In all cases, calibrated Type-T thermocouples (Omega Engineering, Inc.; Stamford, CT) were placed inside vials to ensure accurate temperature monitoring.

### 2.5. Lethal Temperature (LTemp)

Vials containing fed or unfed adults or eggs were placed in the GC oven and the temperature was allowed to increase at a constant rate of 0.06 °C/min from a starting temperature of 30 °C. Vials (n = 36) containing adults were randomly assigned to temperatures of 30, 35, 40, 43, 44, 45, 46, 48, 50 or 55 °C. Vials (n = 16) containing eggs were randomly assigned the same temperatures, except 44 and 46 °C. The experiment was run in six trials for adults and three trials for eggs because of the large number of individuals required. There were at least two to six reps per temperature. Control vials containing adults (n = 36 vials) or eggs (n = 16) were simultaneously placed in the CEC and maintained at room temperature (23 °C). As each treatment temperature was attained, the assigned vial was removed from the GC oven along with its corresponding control vial from the CEC. All vials were then held for 24 h at 23 °C, after which time adult mortality was recorded. Adult mortality was determined as the lack of movement after prodding with forceps. Egg mortality was determined as the failure to produce live nymphs and was recorded after a 2-wk interval.

### 2.6. Lethal Time at Set Temperatures (LTime)

Lethal time experiments proceeded similarly to LTemp, with the exception that vials were randomly assigned to exposure times of 2, 10, 20, 40, 60, 90 or 120 min at each temperature that included: 30, 35, 40, 43, 45, 48, 50 and 55 °C. There were 2 vials per time and temperature for adults (n = 112 vials) and 1 vial per time and temperature for eggs (n = 56 vials). A second experiment was conducted at 45 °C to determine the extended survival of eggs compared to adults. Exposure times were 4, 6, 8 and 12 h in addition to the 2–120 min range mentioned previously. Control vials containing fed or unfed adults or eggs were simultaneously set aside at room temperature. Mortality of adults and eggs was assessed as previously described.

### 2.7. Data and Statistical Analyses

**Figure 1:**
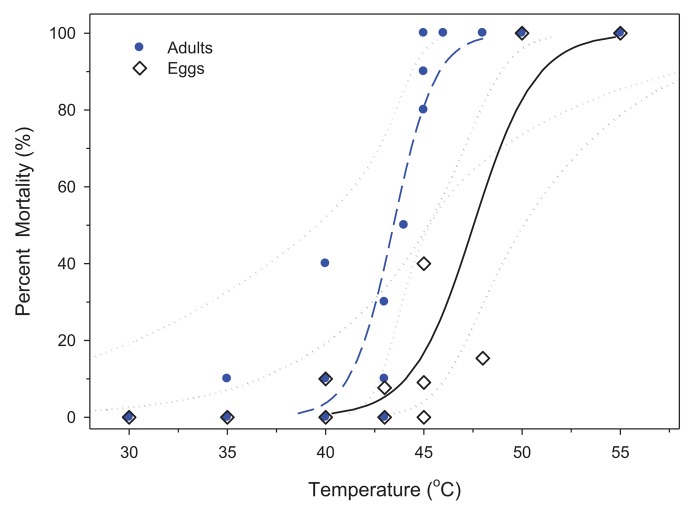
Mortality of bed bug adults ( 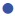) and eggs (◊) when exposed to different final temperatures after a temperature ramp of 0.06 °C/min. Logistic regression for adults (y = 0.943 Log_10_(x) −40.96; 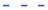) and eggs (y = 0.636 Log_10_(x) −30.21; ―) are displayed with their respective 95% CLs ( 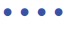 and ····).

For LTemp, mortality and temperature data were evaluated via Proc Probit with Logistic option [16]. This enabled calculation of LTemp estimates with 95% confidence intervals (CI) at 50 and 99% mortality. LTemp for eggs and adults were compared based on the LTemp_50,99_ values. Confidence intervals were included in graphs as limits for the central estimators. Bed bug mortality in control vials was negligible; it was below 10% during all experiments. In both stages of bed bugs tested, the goodness of fit test from the Probit (Logistic) analysis was significant (*P* = 0.001) and variances were multiplied by a heterogeneity factor. Confidence limits were calculated using a t-value of 2.03 for adults and 2.20 for eggs (SAS PROC Probit, [16]). Despite excess variability, the data did not show a systematic departure from the Logistic model (Figure 1).

For LTime, data were first graphed to determine the presence of a sigmoidal mortality relationship within 120 min. Threshold temperature was defined as the temperature where mortality occurred within 2 h. Temperatures were considered ineffective for heat treatment when there was <100% mortality of bed bugs after 2 h. When the majority of bed bugs died during the temperature ramp phase and prior to the assigned temperatures, this condition was considered above the threshold temperature. Conditions above the threshold temperature will control bed bugs, but there is a risk of increased costs in time and energy from unnecessary heating and holding of high temperatures.

## 3. Results and Discussion

For LTemp, mortality was first observed at 40 °C and reached 100% mortality at 50 and 55 °C (Figure 1). The LTemp_50,99_ between fed and unfed adults was not significantly different, so the data were combined (data not shown). For adults, the LTemp_50,99_ was 43.5 °C and 48.3 °C, respectively (Table 1). Eggs had a higher LTemp_50,99_ of 47.5 °C and 54.8 °C, respectively (Figure1, Table 1).

**Figure 2:**
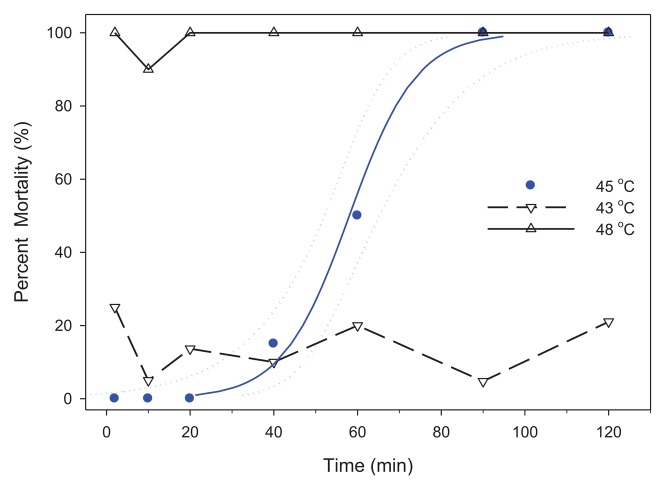
Threshold temperature for mortality of adult bed bugs ( 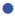); Logistic regression 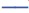, CL 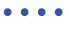) within 2 h of attaining a target temperature of 45 °C. The other two lines indicate mortality of bed bugs above and below this threshold temperature. Regression characteristics for the line at 45 °C was y = 0.125 Log10(x) −7.25.

At temperatures below LTemp_99_, exposure time becomes important. For adults, the threshold temperature was 45 °C and the LTime_50,99_ was 58.0 min and 94.8 min, respectively (Figure 2, Table 2). Similar to LTemp_50,99_, the LTime_50,99_ of fed and unfed adults was not significantly different and the data were combined (data not shown).

**Figure 3:**
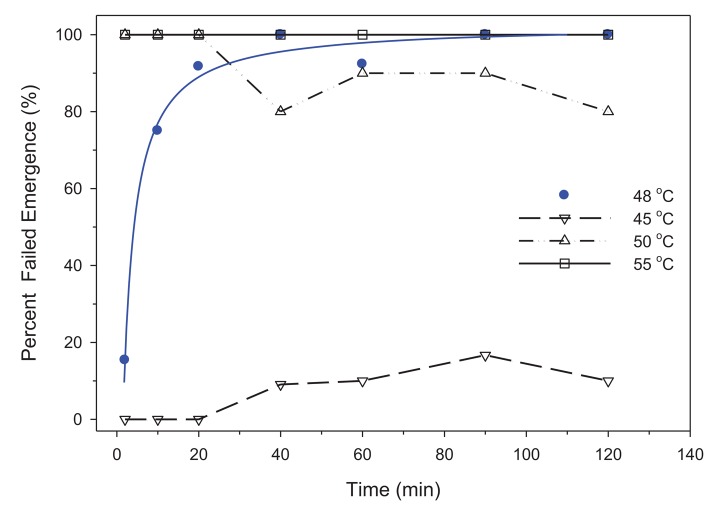
Threshold temperature for bed bug eggs that failed to emerge ( 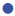; Hyperbola regression 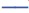) within 2 h of attaining a target temperature of 48 °C. The other three lines indicate the mortality response of eggs above and below this threshold temperature. Line characteristics for the hyperbola regression are: y = −1.16 + 2.19x/(1.33 + x); r^2^ = 0.99.

The threshold temperature was 48 °C for eggs. Failed emergence of eggs was <20% for temperatures below 48 °C and with 2 h of exposure (Figure 3). Exposure of eggs to 48 °C resulted in a hyperbola relationship for failed emergence rather than the standard sigmoidal relationship used by Logistic analysis. As a result, a three-parameter hyperbolic regression produced the following equation with an r^2^ = 0.99 (n = 7):

(1) y=−1.16+2.19x/(1.33+x)

The LTime_50,99_ for eggs at 48 °C was 4.2 and 71.5 min, respectively. Unfortunately the confidence interval for a hyperbolic regression is not conducive for reversed x | y confidence intervals. Interestingly, a few nymphs (n = 6) were found in vials exposed to 50 °C at 40–120 min (Figure 3); however, none of these nymphs were alive and it is unclear when these nymphs had emerged. We suspect that the eggs may have been produced early during the 5 d egg collection period and these eggs were close to hatching when exposed to the heat treatment. No nymphs emerged at 55 °C.

**Figure 4:**
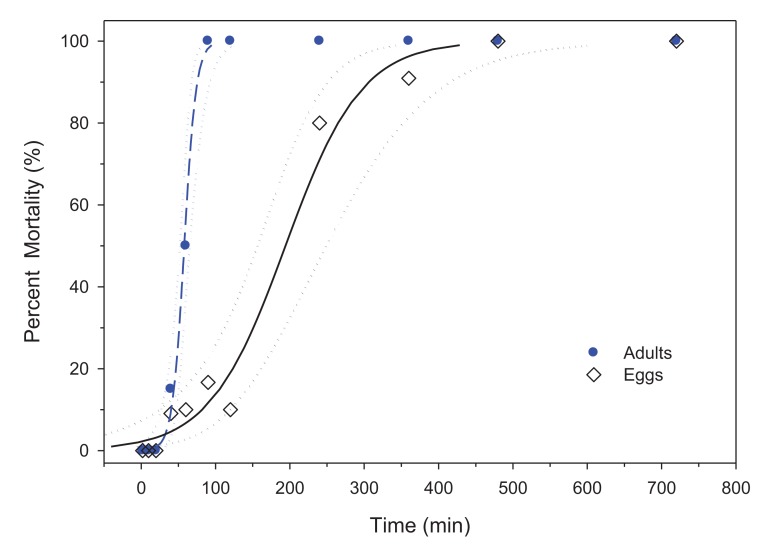
Mortality of bed bug adults ( 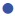) and eggs (◊) when exposed to 45 °C after a temperature ramp of 0.06 °C/min. Logistic regression for adults ( 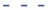) and eggs (―) are displayed along with 95% CI ( 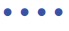 and ····). Regression characteristics were y = 0.125 Log_10_(x) −7.25 for adults and y = 0.0196 Log_10_(x) −3.80 for eggs.

In this study, bed bug eggs survived 120 min at 45 °C with minimal mortality, while adults failed to survive beyond the 2-h exposure time (LTime_99_ = 94.8 min). To determine the effects of extended exposure, adults and eggs were exposed to 4, 6, 8 and 12 h in addition to previous time points. With significantly longer survival times compared to adults at 45 °C, the LTime_50,99_ for eggs was 194.1 and 428.5 min, respectively (Table 2, Figure 4).

Developing alternatives to insecticidal treatments against bed bugs requires a balance between obtaining complete mortality while minimizing labor, equipment and energy costs. Knowledge of the effects of elevated temperatures on bed bugs is needed to ensure that heat treatments are both cost effective and practical. We identify the minimum lethal temperatures (LTemp) required to immediately kill the egg and adult life stages of bed bugs. Furthermore, we report the time (LTime) required to kill eggs and adults, should the minimum lethal temperature not be achieved. Achieving appropriate LTemp or LTime estimates will avoid heat refugia and treatment failure.

In this study, temperatures of 48 °C for 71.5 min or >50 °C (0 min) are required for whole-room heat treatments. These temperatures are substantially greater than previously published values. Mellanby [10] reported 100% mortality for 1 and 24 h exposures at 45 and 41 °C for eggs; and 44 and 40 °C for adults. Pereira *et al.* [9] indicated mortality of adults required 100 min at 41 °C, 10 min at 45 °C and 1 min at 49 °C. Adult mortality in 1 min at 49 °C is similar to our results (Figure 2). However, our findings demonstrated that bed bug eggs are much more resistant to high temperatures than are adults. For control of bed bugs, the practical temperature often cited is 45 °C [1], but we demonstrated that eggs were viable for up to 7 h when exposed to 45 °C (Figure 4) and 71.5 min at 48 °C (Figure 3).

The discrepancy in our results towards higher LTemp and LTime may be attributed to different methodologies. Mellanby [10] and Pereira *et al.* [9] used a step-function approach [17] where bed bug mortality was recorded after rapid transfer from a baseline temperature to an elevated one (e.g., immersion in heated water bath). We employed a ramp-function approach where bed bugs were exposed to a linear rate of temperature increase more representative of a whole-room heat treatment. Different rates of heating can have a substantial impact on insect survival. For example, a 4.3-fold increase in LTemp95 was demonstrated for codling moths when they were heated at a slower rate [18]. Further research should evaluate mortality with different rates of heating and different initial temperatures to further refine temperature requirements for control of bed bugs. This additional research might explain the discrepancies found between the current and previous studies.

Previous exposure temperatures and increased synthesis of heat shock proteins in response to thermal stress may have an impact on the survival of bed bugs. Benoit *et al.* [19] did not detect enhanced survival with heat acclimation through Rapid Heat Hardening (RHH). However, bed bug survival at higher temperatures than previously reported may indicate that some physiological changes occurred during a slower heating process. It would be interesting in future studies to evaluate the response of RHH in bed bugs via the ramp function approach employed in our study. The percent survival indicated in Benoit *et al.* [19] did agree with the temperature-mortality estimates in our study, as some adults survived beyond the mortality curves presented in Pereira *et al.* [9].

The goal of our experiment was to provide temperature and time estimates under a so called “worst-case habitat scenario”. Bed bugs were pre-exposed to slightly elevated temperatures that are within the range for human habitation. We assumed that the rate of temperature increase may be slowed by insulated areas, and/or there may be cases where the use of under-capacity heaters requires additional time to achieve target temperatures. In preliminary monitoring of whole-room heat treatments, temperatures increased at a rate dependent on insulative qualities of materials, distance from the heat source and the use of circulation fans. The slowest rate of heating encountered was 0.06 °C/min (3.6 °C/h) and the highest rate was 0.2 °C/min (12 °C/h). Using oil emersion heaters contained within an insulated box, Pereira *et al.* [9] generated similar heating rates (0.08–0.2 °C/min). Complexity within the living space and different rates of heating may have a marked effect on a bed bug's ability to withstand high temperatures. Through the use of temperature sensors, practitioners can determine which areas have reached lethal conditions (>50 °C) and which areas require continued exposure times (71.5 min at 48 °C) to ensure control. Future studies should continue the use of a ramp-function approach to evaluate the potential for RHH and to evaluate bed bugs pre-conditioned at a wider temperature range.

## 4. Conclusions

Our findings show that the LTemp_99_ estimate was 48.3 and 54.8 °C for adults and eggs, respectively. If these temperatures are not achieved, then exposure time becomes important. For eggs, 48 °C for at least 71.5 min was necessary for complete lethality (Figure 3). Eggs may hatch at 50 °C, but no nymphs survived indicating that this temperature is likely the minimal level for immediate mortality. The Probit for immediate mortality of eggs was estimated at 54.8 °C (51.5, 70.0), but this may be an over-estimate as a result of a low mortality at 48 °C which shifted the curve to the right and exaggerated variability (Figure 1). Survival was not observed at temperatures >50 °C in either the Probit or timed trials. Based on these results, eggs are more heat resistant than adults, and minimum exposure times for a whole-room heat treatment should be 48 °C for 71.5 min or cessation once ≥50 °C is achieved in all sites where bed bugs may hide.

## Figures and Tables

**Table 1:** Lethal Temperature (LTemp) estimates for 50 and 99% mortality of bed bug adults and eggs.

**Stage**	**Mortality Temperature Estimates**			

	**LTemp_50_**		**LTemp_99_**	
	**°C**	**(95% CI)**	**°C**	**(95% CI)**
Adults	43.5	(39.5, 45.3)	48.3	(46.0, 75.5)
Eggs	47.5	(45.2, 50.1)	54.8	(51.5, 70.0)

**Table 2:** Lethal time estimates for 50 and 99% mortality of bed bugs exposed to temperatures below the lethal temperature of 48.3 °C for adults and 54.8 °C for eggs.

**Stage**	**Mortality Time Estimates**			

	**LTime_50_**		**LTime_99_**	
	**min**	**(95% CI)**	**min**	**(95% CI)**
Adults at 45 °C	58.0	(52.0, 65.2)	94.8	(82.0, 125.3)
Eggs at 45 °C	194.1	(156.4, 249.0)	428.5	(344.0, 606.3)
Eggs at 48 °C	4.2	(--, --) a	71.5	(--, --)

a - CI not estimatable.

## References

[1] Usinger R. (1966). Monograph of Cimicidae (Hemiptera-Heteroptera) (The Thomas Say Foundation).

[2] Paul J., Bates J. (2000). Is infestation with the common bedbug increasing?. Br. Med. J..

[3] Doggett S., Geary M., Russell R. (2004). The resurgence of bed bugs in Australia: With notes on their ecology and control. Environ. Health.

[4] Hwang S.W., Svoboda T.J., de Jong I.J., Kabasele K.J., Gogosis E. (2005). Bed bug infestations in an urban environment. Emerg. Infect. Dis..

[5] Romero A., Potter M.F., Potter D.A., Haynes K.F. (2007). Insecticide resistance in the bed bug: A factor in the pest's sudden resurgence?. J. Med. Entomol..

[6] Moore D.J., Miller D.M. (2006). Laboratory evaluations of insecticide product efficacy for control of *Cimex lectularius*. J. Econ. Entomol..

[7] Kells S.A. (2006). Nonchemical control of bed bugs. Am. Entomol..

[8] Pinto L.J., Cooper R, Kraft S.K. (2007). Bed Bug Handbook: The Complete Guide to Bed Bugs and Their Control.

[9] Pereira R.M., Koehler P.G., Pfiester M., Walker W. (2009). Lethal effects of heat and use of localized heat treatment for control of bed bug infestations. J. Econ. Entomol..

[10] Mellanby K. (1935). A comparison of the physiology of the two species of bed-bug which attack man. Parasitology.

[11] Neven L.G. (2000). Physiological response of insects to heat. Postharvest Biol. Technol..

[12] Doggett S.L., Geary M.J., Russell R.C. (2006). Encasing mattresses in black plastic will not provide thermal control of bed bugs, *Cimex* spp. (Hemiptera: Cimicidae). J. Econ. Entomol..

[13] Lester P.J., Greenwood D.R. (1997). Pretreatment induced thermotolerance in lightbrown apple moth (Lepidoptera: Tortricidae) and associated induction of heat shock protein synthesis. J. Econ. Entomol..

[14] Olson J.F., Moon R.D., Kells S.A. (2009). Off-host aggregation behavior and sensory basis of arrestment by *Cimex lectularius* (Heteroptera: Cimicidae). J. Insect Physiol..

[15] Montes C., Cuadrillero C., Vilella D. (2002). Maintenance of a laboratory colony of *Cimex lectularius* (Hemiptera: Cimicidae) using an artificial feeding technique. J. Med. Entomol..

[16] SAS Institute Inc. (2008). SAS/STAT® 9.2 User's Guide.

[17] Clarke K.U., Rose A.H. (1967). Insects and Temperature. Thermobiology.

[18] Neven L.G. (1998). Effects of heating rate on the mortality of fifth instar codling moth. J. Econ. Entomol..

[19] Benoit J.B, Lopez-Martinez G., Teets N.M., Phillips S.A., Denlinger D.L. (2009). Responses of the bed bug, *Cimex lectularius*, to temperature extremes and dehydration: Levels of tolerance, rapid cold hardening and expression of heat shock proteins. Med. Vet. Entomol..

